# Analysis of Primary Cilium Expression and Hedgehog Pathway Activation in Mesothelioma Throws Back Its Complex Biology

**DOI:** 10.3390/cancers14215216

**Published:** 2022-10-25

**Authors:** Marcella Barbarino, Maria Bottaro, Laura Spagnoletti, Maria Margherita de Santi, Raffaella Guazzo, Chiara Defraia, Cosimo Custoza, Gabriella Serio, Francesco Iannelli, Matilde Pesetti, Raffaele Aiello, Diletta Rosati, Edoardo Zanfrini, Luca Luzzi, Cristiana Bellan, Antonio Giordano

**Affiliations:** 1 Department of Medical Biotechnologies, Siena University, 53100 Siena, Italy; 2 Sbarro Institute for Cancer Research and Molecular Medicine, Center for Biotechnology, College of Science and Technology, Temple University, Philadelphia, PA 19122, USA; 3 Department of Emergency and Organ Transplantation-DETO, University of Bari, G. Cesare 1 Sq., 70121 Bari, Italy; 4 Toma Institute Srl, Via Cesare Rosaroll 24, 80139 Napoli, Italy; 5 Department of Medicine, Surgery and Neurosciences, Siena University Hospital, 53100 Siena, Italy

**Keywords:** primary cilium, mesothelioma, pleural, hedgehog signaling, GLI1

## Abstract

**Simple Summary:**

Primary cilium (PC) is a solitary organelle protruding from the cellular membrane of almost all mammalian cells. It transduces signals from external to intracellular compartments regulating many physiological pathways. Dysfunction in PC can lead to a number of pathologic conditions including cancer. Among the pathways transduced by PC, the Hedgehog (Hh) signaling is the focus of recent clinical studies. Malignant pleural mesothelioma (MPM) is a cancer of the membranes covering the lungs with poor prognosis and limited therapeutic options. Although Hh is known to be over-activated in MPM, it responds poorly to smoothened (SMO)/Hh inhibitors. We investigated for the first time the status of PC in MPM tissues, demonstrating intra- and inter-heterogeneity in its expression. We also correlated the presence of PC with activation of the Hh pathway, providing uncovered evidence of the co-existence of a PC-independent regulation of the Hh signaling in MPM.

**Abstract:**

The primary cilium (PC) is a sensory organelle present on the cell surface, modulating the activity of many pathways. Dysfunctions in the PC lead to different pathologic conditions including cancer. Hedgehog signaling (Hh) is regulated by PC and the loss of its control has been observed in many cancers, including mesothelioma. Malignant pleural mesothelioma (MPM) is a fatal cancer of the pleural membranes with poor therapeutic options. Recently, overexpression of the Hh transcriptional activator GL1 has been demonstrated to be associated with poor overall survival (OS) in MPM. However, unlike other cancers, the response to G-protein-coupled receptor smoothened (SMO)/Hh inhibitors is poor, mainly attributable to the lack of markers for patient stratification. For all these reasons, and in particular for the role of PC in the regulation of Hh, we investigated for the first time the status of PC in MPM tissues, demonstrating intra- and inter-heterogeneity in its expression. We also correlated the presence of PC with the activation of the Hh pathway, providing uncovered evidence of a PC-independent regulation of the Hh signaling in MPM. Our study contributes to the understanding MPM heterogeneity, thus helping to identify patients who might benefit from Hh inhibitors.

## 1. Introduction

Primary cilium (PC) is a microtubule-based non-motile sensory organelle located on the surface of all mammalian cells that, similar to an antenna, gathers extracellular signals through different transmembrane receptors [1]. Many different pathways start on PC, with Hedgehog (Hh), Platelet-Derived Growth Factor (PDGF), Transforming Growth Factor-beta (TGFβ), NOTCH, Hippo, mammalian target of rapamycin (mTOR), and Wingless/Integrated (Wnt) having important roles in the regulation of crucial biological functions, such as development, differentiation, and diseases pathogenesis [2,3,4]. An exhaustive comprehension of all PC functions is far from being achieved, and this remains an active field of research.

PC is lost in a group of developmental genetic disorders, collectively known as ciliopathies, and in a variety of human neoplasms [5], leading to the hypothesis that PC is a tumor-suppressor organelle [6,7,8,9,10,11,12]. However, different cancers retain PC, such as basal cell carcinomas (BCC), pleomorphic adenomas, neuroblastoma, colorectal tumors, craniopharyngioma, a subset of medulloblastoma and gastrointestinal stromal tumors [13,14,15,16,17,18], supporting the recent hypothesis that PC has tissue- and cancer-specific roles.

Among the pathways regulated by PC, the Hedgehog (Hh) has gained the attention of the scientific community for its function as a regulator of the onset and progression of many cancers [19,20].

Hh is an intricate but highly regulated signaling cascade regulating many crucial developmental steps and, under physiological conditions, is repressed in developed tissues [21]. Hh is activated by the binding of extracellular ligands to specific receptors located in the PC which initiate a cascade of signals that ultimately leads to the transcription of target genes. The three ligands, Sonic Hedgehog (Shh), Indian Hedgehog (Ihh), and Desert Hedgehog (Dhh) are secreted in both an autocrine and paracrine manner and, by binding to the Hh inhibitory protein PTCH1 localized to the base of PC [22], they allow the release of the G-protein-coupled receptor smoothened (SMO) and subsequent activation of Glioma-associated oncogene-1 and -2 transcription factors (GLI1 and GLI2). Then, GLI transcription factors translocate to the nucleus and transcribe target genes (for a complete review of the activation and repression of GLI transcription factors, see ref [21]). Among them, the transcriptional targets PTCH1 and HHIP [23] function as negative regulators by binding Hh ligands.

This ligand-mediated activation of Hh is called the canonical SMO/Hh pathway and depends on the presence of PC.

In cancer, the mechanisms underlying the aberrant activation of canonical Hh are mainly due to the loss of PTCH-1 and -2 repressors, to the loss of GLI-inhibitory function of Suppressor of Fused homolog (SUFU), to activating mutation in SMO, or overexpression of pathway activators (SMO, GLI1, GLI2, Hh ligands) [24]. Deregulation of SMO/Hh signaling makes tumors resistant to SMO inhibitors (SMO-i) [25].

Aberrant Hh activation in cancer can also arise through non-canonical/SMO independent signaling linked to a number of different oncogenic pathways, such as PI3K/AKT, RAS-RAF-MEK-ERK signaling, KRAS constitutive activation, and TGF-beta. The intricate network of signals that activate Hh in a non-canonical way was recently reviewed by Pietrobono et al. [26].

In some cancers, inhibition of the canonical SMO/Hh pathway has been successful, leading to approval by the Food and Drug Administration (FDA) in 2012 and the European Medicines Agency (EMA) in 2013 of the first SMO inhibitor Vismodegib for the treatment of basal cell carcinoma (BBC), encouraging clinical trials in other cancers.

Although rare, malignant pleural mesothelioma (MPM) is the most common cancer of the pleura, with limited therapeutic options and a dismal prognosis. Three histological subtypes of MPM can be distinguished, having different incidence and prognosis. Epithelioid MPM accounts for the majority of cases and correlates with a better prognosis, while sarcomatoid, desmoplastic and mixed (biphasic) histotypes are the most aggressive and correlated with a worst prognosis [27].

In recent years, limited progress has been made in the treatment of MPM mainly due to its intrinsic heterogeneity, the lack of markers for early diagnosis, and the high immunosuppressive microenvironment that characterizes this tumor [28,29]. First-line therapy is a combination of platinum agents plus folate antimetabolites with the optional addiction of Bevacizumab [30]. Recently, nivolumab plus ipilimumab was introduced for the treatment of selected unresectable mesothelioma [31]. However, due to the lack of markers for patient stratification, personalized therapies remain an urgent need.

Many studies have shown that the Hh pathway is deregulated in most MPM [32,33,34] and that high levels of downstream transcriptional targets GLI1 and SMO are correlated with a poor prognosis [33,35]. However, the expression of Hh components in MPM is not correlated with the expression of their upstream stimuli Shh and SMO [32] and there is a lack of direct evidence linking GLI activity to Hh. Accordingly, the direct inhibition of GLI1 in MPM demonstrated greater efficacy then SMO-i [32,35,36,37]. Unfortunately, no direct-acting GLI1 inhibitors have already entered clinical trials [38].

Despite these results, only two in vitro studies have investigated the presence of PC in MPM as a marker of SMO/Hh activation [37,39,40]. This, therefore, remains an important issue for the selection of patients who may respond to SMO inhibitors and for understanding the mechanisms underlying resistance to this class of drugs. In fact, unlike other cancers, mutations in the components of the pathway are rare in MPM and cannot explain its resistance to treatments [31,38].

In light of the good clinical results achieved with SMO-i in other tumor types, and since MPM is a highly heterogeneous cancer, we believe that this class of compounds can be repurposed to treat a subset of MPM. However, as with other targeted therapies, proper predictive biomarkers are needed.

On these premises, here we investigated the presence of PC in MPM tissues and in a panel of primary cell lines, correlating its presence with the Hh activation. Our finding demonstrated a high heterogeneity in PC expression, and its loss is a frequent event in the more aggressive histotypes.

Our study provided uncovered evidence that the Hh pathway in MPM can also be activated by non-canonical/PC-independent mechanisms explaining, at least in part, the lack of response of MPM to SMO/Hh inhibitors upstream of GLI1.

Furthermore, our analysis adds new insights into the role of PC expression in cancer. Although our findings need to be confirmed in a larger cohort, this study identified a new cellular marker of MPM heterogeneity that could be correlated to MPM progression, providing another puzzle piece in understanding the complexity of MPM biology, in an attempt to offer personalized treatments for this tumor in the near future.

## 2. Materials and Methods

### 2.1. Cell Lines and Culture Conditions

Mesothelioma cell lines were isolated from patients who underwent multiple thoracoscopic biopsies at the Thoracic Surgery Unit of the Siena university Hospital (Siena, Italy) prior and/or after chemotherapy, between 2016 and 2022. All specimens analyzed were from patients diagnosed for pleural mesothelioma with their written informed consent. Two patients with pleurisy and two with reactive mesothelial hyperplasia were included in the non-cancer group. Human investigations were performed with approval from the Research Ethics Committee (Comitato Etico Regione Toscana-Area Vasta Sud Est) approval (#CCMESOLUNG, #Mi-PP). The study conformed to the standards of the Declaration of Helsinki. All cases were classified as mesothelioma according to the 2021 morphologic and immunophenotypic WHO classification [27].

Tissues and pleural effusions were processed as previously described [41]. Briefly, the samples were transported to the laboratory for primary cell culturing within 30 min of collection. The solid tissues were minced into small pieces, 1–3 mm, and then incubated in complete medium supplemented with collagenase type I from Clostridium histolyticum (Thermo Fisher Scientific, Waltham, MA, USA, Cat #17100017) at 200 U/mL concentration for 1 h to digest collagen and release tumor cells. Pleural effusions were centrifuged at 400× *g* and washed twice in PBS. Macrophages, red blood cells and lymphocytes were the main contaminants. To avoid their interference in the analysis, all the cell lines were used after the 5th passage.

The mesothelial origin of patients-derived cultures was assessed by immunohistochemical analysis (IHC) for a panel of markers (WT-1, α-SMA, CD31, CD34). The cell lines were also analyzed by transmission electron microscopy (TEM) for the presence of microvilli on the cellular membrane. Briefly, the cells were fixed in 2.5% cacodylate-buffered glutaraldehyde, post-fixed in buffered 1% osmium tetroxide, dehydrated, and embedded in epoxy resin. Ultrathin sections were double-stained with uranyl-acetate replacement stain (UAR) and lead citrate and examined with a Philips 208S Transmission Electron Microscope.

Clinical information on patients (age, gender, diagnosis, and treatment) from whom the cell lines have been established are summarized in Table S1. Non-tumoral mesothelial cell lines were established from the pleural effusion of non-oncologic patients for whom the final diagnosis was negative for MPM.

HMC7 cells were immortalized using the Lentiviral vectors pLenti-hTERT-Neo (Applied Biological Materials, Richmond, BC, Canada, Cat #LV622). Cells were transduced according to the manufacturer’s instructions. Briefly, 5.0 × 10^3^ cells/cm^2^ were plated in six-well culture plates and incubated at 37 °C, 5% CO_2_ until they reached 65–70% confluence. Afterwards, medium was replenished with pLenti-hTERT viral suspension and fresh medium (ratio 1:1) in the presence of 8 μg/mL polybrene.

Cells were then incubated overnight at 37 °C, 5% CO_2_. After 24 h, the medium was changed, and cells were left in culture for three days. At 72 h post-transduction, cells were incubated in their regular growth medium containing Neomycin (10 μg/m) to select for stable hTERT transduced cells. Selection was carried out until the cultures were devoid of non-resistant cells (<14 days) and surviving cells were further expanded in standard medium and routinely passaged.

MPM patient’s derived cell lines and HMC7 cells were cultured in Medium 199 (Euroclone, Pero, Italy, Cat#ECB2056L), supplemented with 2 mmol/L L-glutamine (Euroclone, Cat #ECB3000D), 100 U/mL penicillin, 100 μg/mL streptomycin (Euroclone, Cat# ECB3001D), 10% FBS (Euroclone, Cat#0180L). LP-9 cells were from Coriell Institute (Camden, NJ, USA, Cat# AG07086, RRID:CVCL_E109), and were cultured according to the manufacturer’s instruction.

Non-malignant primary mesothelial cells HMC12 and HMC13 were grown with the addition of 20 ng/mL hr-EGF (Sigma-Aldrich, ST. Louis, MO, USA).

MSTO-211H (RRID:CVCL_1430) and NCI-H2052 (RRID:CVCL_1518) cells were from American Type Culture Collection (ATCC, Manassas, VA, USA) and cultured in RPMI supplemented with 2 mmol/L L-Glutamine (Euroclone), 100 μg/mL Streptomycin (Euroclone), 10% FBS (Euroclone).

All cell lines were grown at 37 °C in a humidified atmosphere with 5% CO_2_, passaged every 3–5 days, and routinely tested for mycoplasma contamination using MycoSPY^®^-PCR Mycoplasma Test Kit (BIONTEX Laboratories, München, Germany) according to the manufacturer’s instructions.

### 2.2. Immunofluorescence (IF)

Cells were seeded on glass coverslips, allowed to attach for 48 h, and then fixed for 10 min in 4% paraformaldehyde, washed in PBS, incubated for 5 min in methanol, and then incubated over-night at 4 °C with rabbit polyclonal anti-Arl13b (Proteintech, Rosemont, IL, USA; Cat #17711-1-AP, RRID: AB_2060867) and anti-GLI1 (Santa Cruz Biotechnology, CA, USA; Cat #sc-515781). The appropriate secondary antibodies were incubated for 45 min at room temperature (anti-mouse Alexa Fluor 555 (Invitrogen, Waltham, MA, USA; Cat #A21422) or anti-Rabbit Fitc (Sigma-Aldrich, St. Louis, MO, USA, Cat #F9887). Nuclei were counterstained with 4′,6-diamidino-2-phenylindole (DAPI).

Unspecific signal was evaluated for each antibody using a control condition without primary antibody and with a non-specific antibody. Images were acquired using the Zeiss Axio microscope (Zeiss Laboratories, White Plains, NY, USA). The proportion of ciliated cells was determined across multiple fields of view for each condition.

### 2.3. Real-Time Reverse Transcriptase-Quantitative PCR (RT-qPCR)

Total RNA was isolated from cell lines using the RNeasy Mini kit (Qiagen, Hilden, Germany Cat #74106) as previously described [42]. RNA concentration was determined using a NanoDrop™ ND-1000 (Thermo Fisher). Complementary DNA was synthesized from 500 ng of RNA using the iScript cDNA Synthesis Kit (Bio-rad, Hercules, CA, USA Cat #1708891BUN) and amplified in the LightCycler™ instrument (Roche Applied Sciences) using SsoAdvanced™ Universal SYBR^®^ Green Supermix (Bio-rad, Cat #1725274) according to the manufacturer’s instructions.

The primers used were from Bio-Rad: *GLI1* Assay IDqHsaCED0043346, *PTCH1* Assay ID qHsaCED0001809, *C-MYC* Assay ID qHsaCID00012921. The housekeeping glyceraldehyde-3-phosphate dehydrogenase (*GAPDH* Assay ID qHsaCED0038674) gene was used to normalize the expression of the genes of interest. Gene expression levels were calculated by the 2^−ΔΔCt^ method [43].

### 2.4. Immunohistochemical Analysis

Formalin-fixed, paraffin-embedded (FFPE) tumor specimens were obtained from the Section of Pathology of the Siena University Hospital, Siena, Italy. Immunohistochemistry staining was performed on 4-μm-thick paraffin sections adjacent to those that had been stained for Haematoxylin & Eosin (H&E). Clinical information about mesothelioma patients is summarized in Table S2.

The primary rabbit polyclonal anti-Arl13b (Proteintech, Cat #17711-1-AP, RRID: AB_2060867) and anti-GLI1 (Santa Cruz Biotechnology, Cat #sc-515781) were used according to the manufacturer’s instructions.

Frequency of cilia was determined by dividing the number of ciliated cells by the total number of nuclei. MPM with PC frequency less than 15% were considered as negative.

The assessment of nuclear GLI1 expression levels included the staining intensity and the percentage of stained cells. The staining intensity was scored as 0 = no staining, 1 = low expression, 2 = moderate expression, 3 = strong expression.

Statistical analysis of the correlation between GLI1 expression was performed using the IBM SPSS Statistics Software, version 25 for Mac. Pearson’s correlation coefficient was used to measures the statistical relationship, or association, between two continuous variables. Kendall’s tau-b correlation coefficient was used for nonparametric measure of the strength and direction of association that exists between two variables measured on at least an ordinal scale.

### 2.5. TCGA Database Analysis

To investigate the association between GLI1 gene expression level and MPM histotype, we performed a differential expression analysis of the single GLI1 gene in the biphasic and epithelioid histotypes of MPM samples. For this analysis, from The Cancer Genome Atlas (TCGA, https://portal.gdc.cancer.gov/; accessed on 28 March 2022), a subset of 19 publicly available samples’ data are retrieved. We selected common features (male sex and tumor stage 3) in order to harmonize the dataset and thus make it more suitable for differential expression analysis using DESeq2. Then, “htseq_count” files (10 epithelial and 9 biphasic) obtained from RNA sequencing experiments were extracted by download and differential expression analysis was performed with the R tool (v4.2.1, https://www.R-project.org/; accessed on 26 August 2022). Using the PlotCounts function, it was possible to investigate differential expression (up or down-regulation of the gene) and significance at the individual gene level. Differential expression is calculated by the DESeq2 package based on the Log2FoldChange value: if Log2FoldChange is <0, the gene is down-regulated; if Log2FoldChange > 0 it will be up-regulated. In the case where Log2FoldChange = 0, the gene will not be differentially expressed [44]. Statistical significance is calculated by the DESeq2 package by estimating the mean and standard deviation in the two groups of samples compared for each gene. From this initial analysis, the algorithm derives *p*-values, which are then corrected with multiple tests to obtain *p*-value-adjusted (padj). Statistical significance is then estimated from the padj for each gene; the lower it is, the higher the significance will be (padj < 0.05). In conclusion, the differential and significative genes were screened using the following criteria: Log2FoldChange ≠ 0 and adjusted *p*-values (padj) < 0.05.

## 3. Results

### 3.1. Malignant Pleural Mesothelioma (MPM) Shows an Heterogeneous Expression of Primary Cilium (PC)

In order to investigate the status of PC in MPM, we performed an exploratory analysis on 28 FFPE pleural tissues (24 mesothelioma and 4 non-oncological specimens).

**Figure 1:**
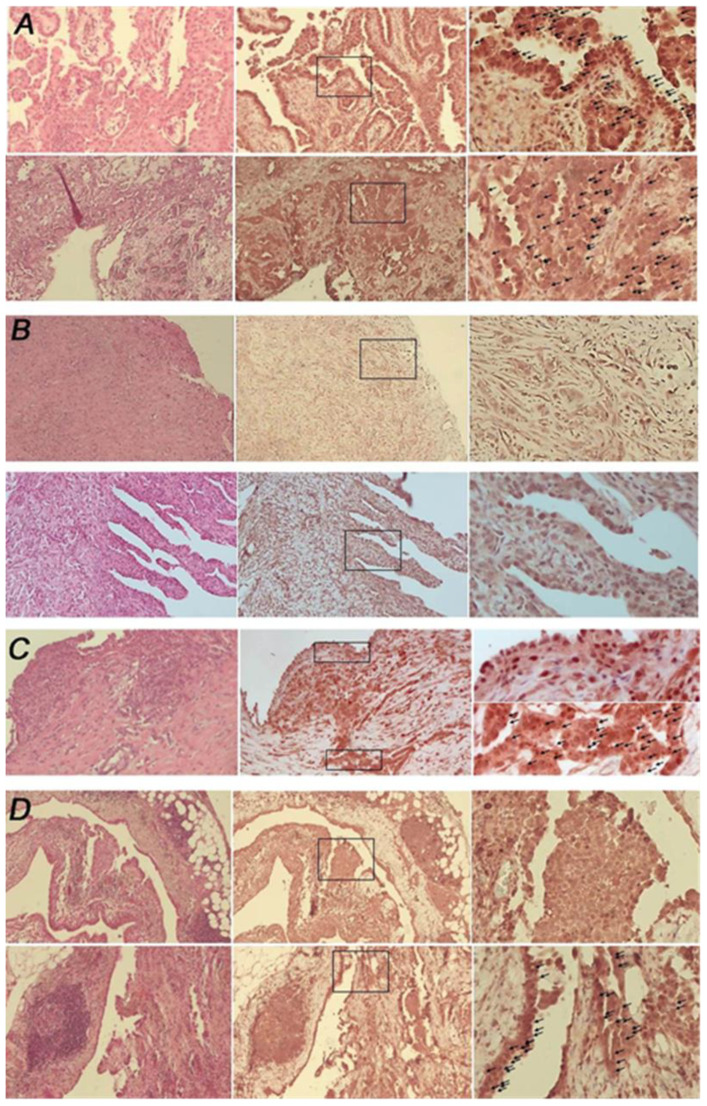
Primary cilium expression in Malignant Pleural Mesothelioma FFPE. (**A**) Representative images of PC positivity from two different epithelioid MPM. (**B**) Representative images of two different PC-negative tissues; upper panel, a biphasic MPM; lower panel, epithelioid MPM. (**C**,**D**) Representative images of PC intra-heterogeneity from two different epithelioid MPM. From left to right: A and B, haematoxylin and eosin staining (magnification 5×), arl13b immunostaining (magnification 5× and 20×); (**C**,**D**), haematoxylin and eosin staining (magnification 5×), arl13b immunostaining (magnification 5× and 40×). Ciliated cells are indicated with arrows.

IHC analysis showed PC expression in seven MPM samples (29.1%) and loss in 13 (54.2%). The latter group includes all the biphasic (*n* = 4) and the desmoplastic (*n* = 1) MPM. The correlation between cilium expression and histology (epitheliomorphic vs. biphasic/desmoplastic mesothelioma) is statistically significant (*p* = 0.020). Four tissues showed heterogeneous intra-tumor expression (16.7%) (Figure 1). Among patients for whom biopsies were available prior to and after chemotherapy (*n* = 4; 8 = samples), only in one patient did we observe a change in PC expression, with a heterogeneous presence of PC prior chemotherapy, and loss after the treatment (data not shown). However, it remains to be ascertained whether this effect is due to a differential response to chemotherapy between PC-positive and PC-negative cells.

All the non-neoplastic cases retain PC. The results are summarized in Table 1.

Then we investigated the expression of PC in eight MPM cell lines (MMP1, MMP4, MMP14, MMP18, MMP21, MMP23, MMP32 and MMP43) and in three non-tumoral mesothelial cell lines (HMC12, HMC13 and HMC7). We included in the analysis one commercial normal mesothelial cell line LP-9, and two long-term MPM cell cultures, MSTO-211H and NCI-H2052.

According to the IHC analysis, all non-tumoral mesothelial cells (HMC7, HMC12, HMC13 and LP-9) expressed PC. Among patients’ derived cell lines, MMP1, MMP4, MMP14, and MMP43 lose the PC, while in MMP18, MMP21, MMP23, and MMP32 the PC expression is retained.

For primary cell lines in which the IHC analysis of matched tissues was available (nine out of eleven cell lines analyzed), we observed the same expression pattern of PC as in the tissue of origin, except for MMP1 which derived from a specimen with an intra-heterogeneous expression of PC. We hypothesize that, during the generation of MMP1 cell line, the cells lacking PC preferentially adapted to in-vitro culture.

**Figure 2:**
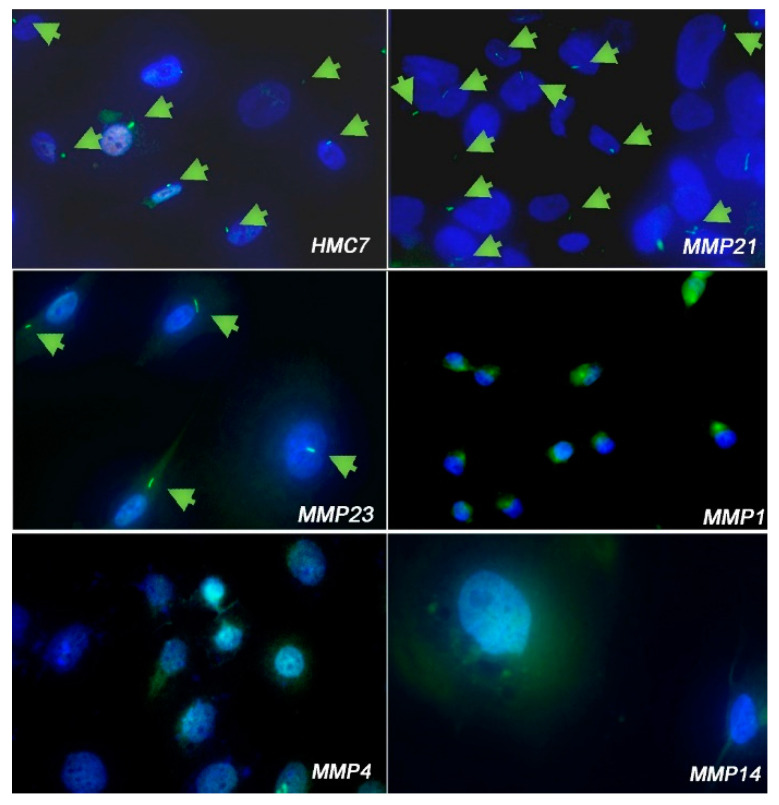
IF analysis of PC expression in selected primary MPM and non-tumoral mesothelial cell lines. PC-positive cell lines: HMC7 normal mesothelial, MMP21, MMP23; PC-negative cell lines: MMP1, MMP4, MMP14. blue: dapi; green: Arl13b. Magnification 20×. Primary cilia are indicated with arrows.

Regarding long term cultures, MSTO-211H cells lose PC, while NCI-H2052 cells express it. The results were summarized in Table 2. Representative images of selected PC- positive and -negative primary MPM cell lines and PC-positive mesothelial cells are shown in Figure 2.

### 3.2. Cell Lines Lacking PC Had Hh/GLI1 Pathway Activated

The Hh is one of the most important oncogenic signaling starting from PC, and one of the top five deregulated pathways in MPM [45] (Supplementary Table S3). For the poor response of MPM to SMO/Hh inhibitors, by qRT-PCR and IF analysis, we explored the activation of Hh in PC-negative MPM cells (MMP1, MMP4 and MSTO-211H), compared to HMC7 mesothelial cells. We also included in the analysis PC-positive MPM cell lines (MMP18, MMP21, MMP23 and NCI-H2052) and normal mesothelial cells LP-9.

**Figure 3:**
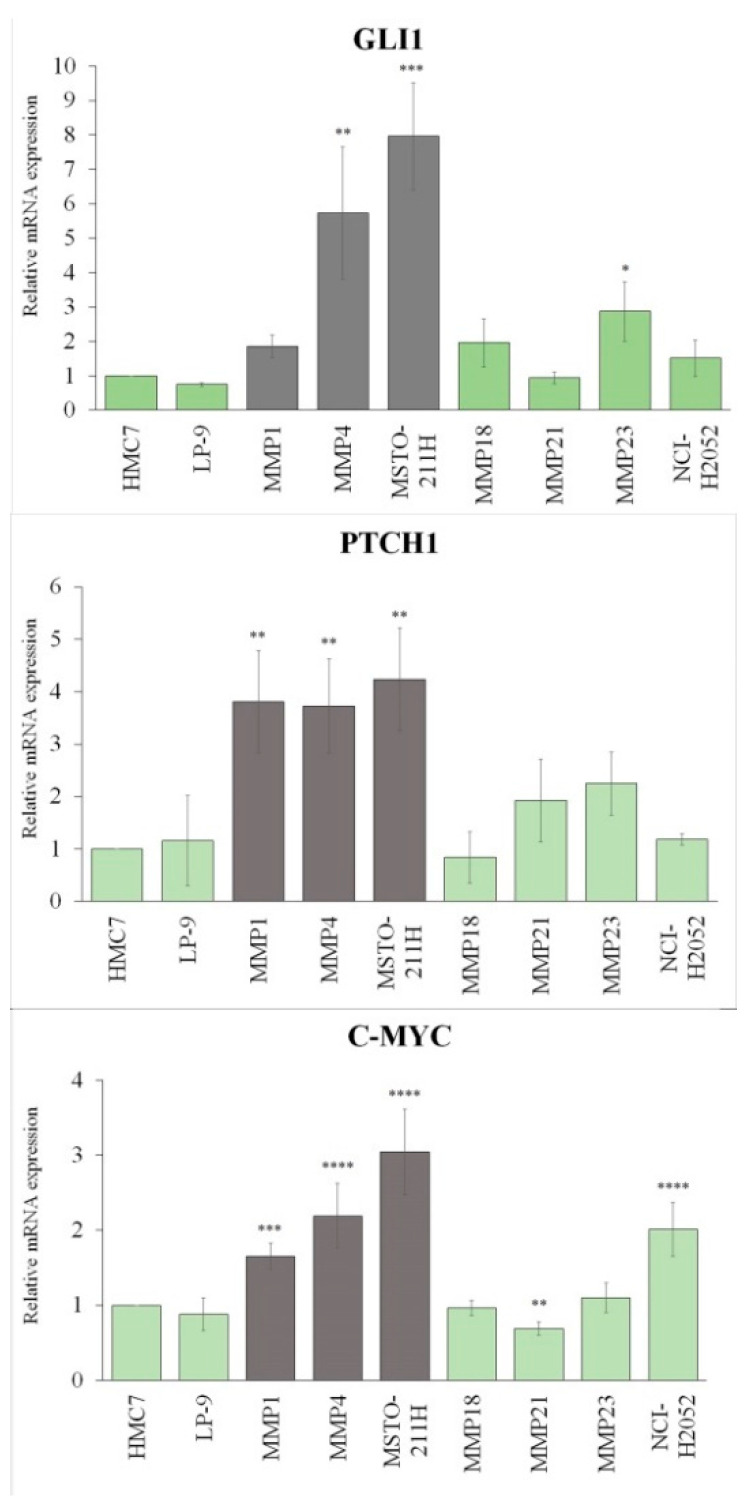
qRT-PCR analysis of *GLI1*, *PTCH1* and *c-MYC.* The expression of Hh-related genes is upregulated in MPM cells loosing PC (gray bars), compared to normal mesothelial cells HMC7. (* *p* < 0.05, ** *p* < 0.01, *** *p* < 0.001, **** *p* < 0.0001).

We analyzed the expression of bona fide Hh/GLI transcription targets *GLI1*, *PTCH1* and *c-MYC*. As shown in Figure 3 and in Table S4, PC-negative cells (gray bars) showed upregulation of all the analyzed genes, even though the *GLI1* transcript in MMP1 did not reach statistical significance. Conversely, in all the PC-positive cells (green bars), the levels of *GLI1*, *PTCH1*, and *c-MYC* are comparable to the those of normal mesothelial cells, with the exception of MMP23, which showed a statistically significant upregulation of *GLI1*, and NCI-H2052, which showed a very significant upregulation of *c-MYC*. However, it should be emphasized that both MSTO-211H and NCI-H2052 cell lines have amplification of the *c-MYC* gene (8q24.1), which is both a target and an activator of *GLI1* [46].

**Figure 4:**
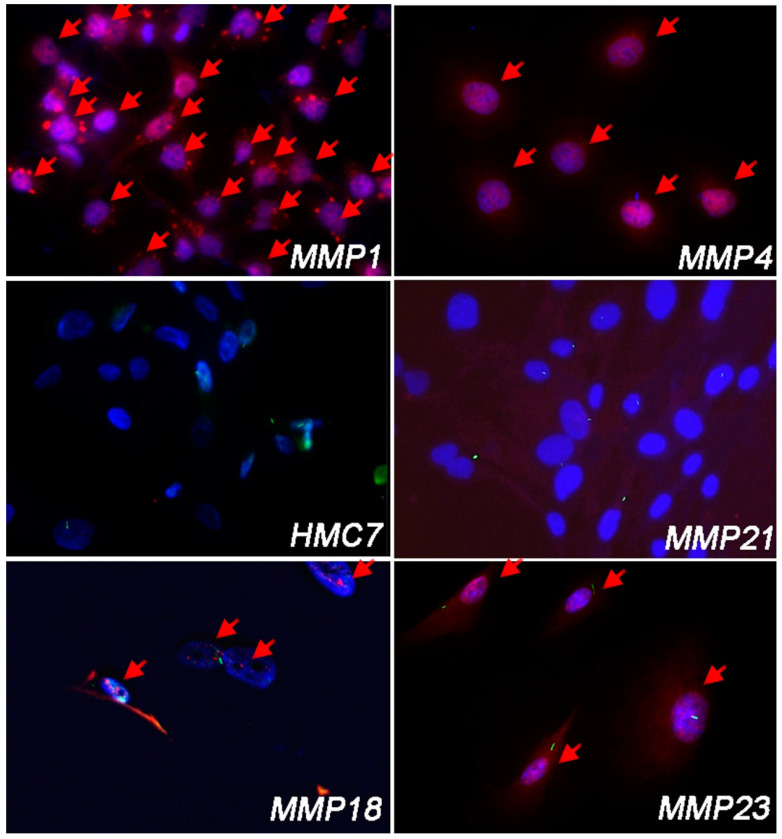
IF analysis of nuclear GLI1 in MPM cell lines and HMC7 normal mesothelial cells. Hh/GLI1 signaling is activated in PC-negative cells MMP1 and MMP4, and in PC-positive cell lines MMP18 and MMP23. Red: GLI1; green: Arl13b; blue: DAPI. The arrows indicate the cells with nuclear GLI1. Magnification 20×.

To confirm these results, we performed IF analysis for GLI1 in primary MPM cells, to assess its nuclear localization. Contrary to qRT-PCR analysis, and thus regardless of PC expression, all MPM cell lines showed nuclear GLI1, except for MMP21 in which GLI1 was undetectable, similar to HMC7 mesothelial cells. Representative images of nuclear GLI1 IF in PC-positive and PC-negative cells are shown in Figure 4.

It is to note that the MMP18 cell line did not show significant upregulation of Hh-related genes by qRT-PCR analysis. However, from the IF analysis, these cells showed the nuclear localization of GLI1, indicating that the transcript levels alone are not a sufficient marker of Hh activation.

### 3.3. GLI1 in MPM Tissues Is Activated Independently from PC Expression

To confirm the results obtained in MPM cell lines, we analyzed GLI1 activation in nineteen MPM (five PC-positive and fourteen PC-negative), according to the availability of tissue specimens.

Positive IHC staining of nuclear GLI1 was observed in twelve out of nineteen MPM samples (63.2%). Among these, three are PC-positive (25%), and nine are PC-negative (75%).

Seven MPM samples (36.8%) did not show nuclear GLI1 staining (Score = 0); of these, two are PC-positive (28.6%), five are PC-negative (71.4%).

The correlation between GLI1 score (0–1 vs. 2–3) and cilium expression was not significant (*p* = 0.373).

**Figure 5:**
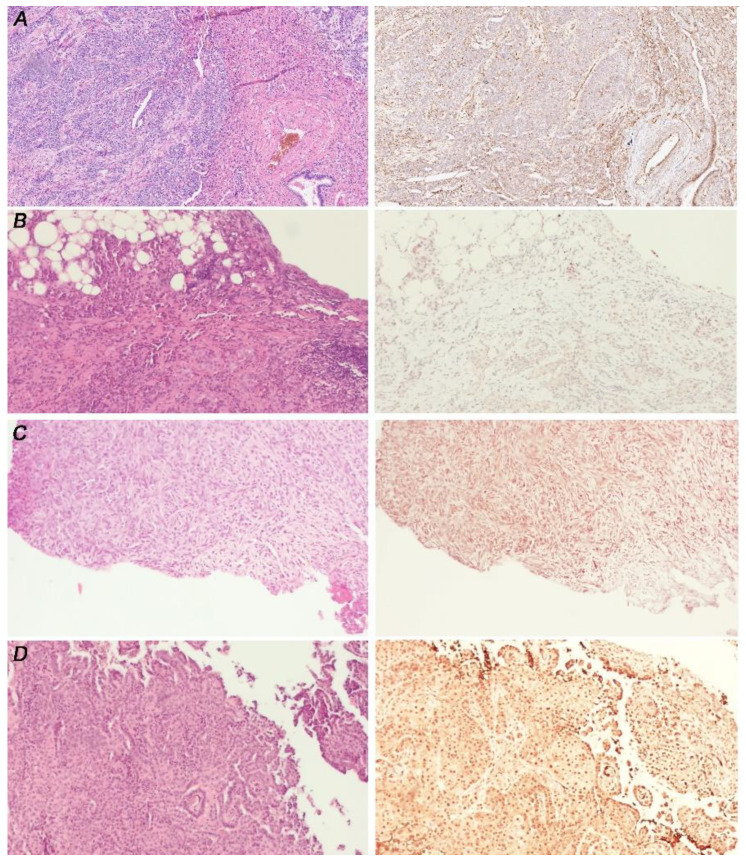
IHC analysis of nuclear GLI1 in MPM FFPE. Magnification 10X. From left to right: haematoxylin and eosin staining, Gli1 immunostaining. (**A**) Epithelioid MPM, negative staining scored 0; (**B**) Epithelioid MPM, faint staining scored 1; (**C**) Biphasic MPM, moderate staining scored 2; (**D**) Epithelioid MPM, strong staining scored 3.

Representative images are shown in Figure 5. The results are summarized in Table 3.

### 3.4. GLI1 Status Does Not Correlated with MPM Histotype

Finally, using the TCGA database, we investigate if *GLI1* is differentially expressed in epithelioid mesothelioma compared to biphasic MPM. We selected a subset of 19 samples with common characteristics (male sex and tumor stage 3).

**Figure 6:**
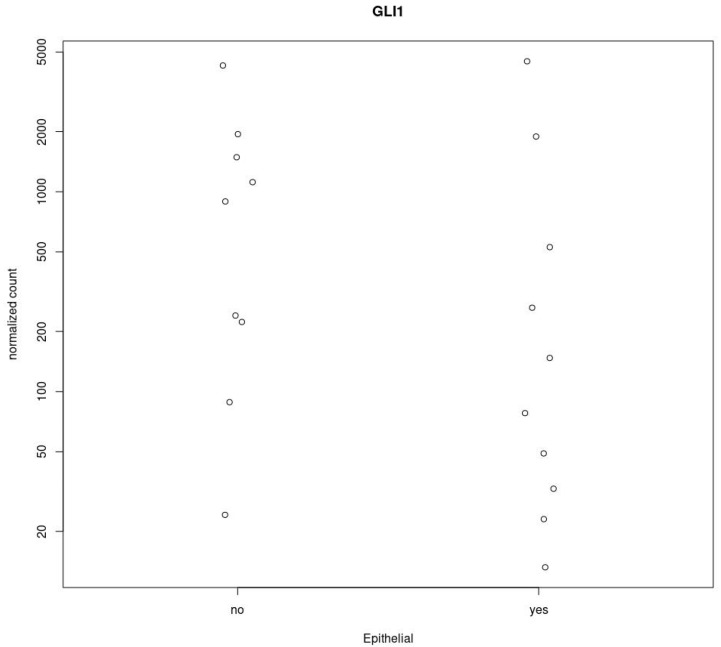
Differential expression analysis of *GLI1* gene between biphasic and epithelioid MPM. The analysis was drawn using the Deseq2-package, PlotCounts. The *GLI1* gene has Log2FoldChange < 0, indicating that the gene is down-regulated in epithelial tissues, but it is not significantly differentially expressed since padj = 0.81. Y axis: normalized count; X axis: Histological subtype. Epithelial = yes; non-epithelial = no.

From this analysis, the *GLI1* gene (Ensembl ID: ENSG00000111087.8) has Log2FoldChange < 0, indicating that the gene is downregulated in epithelial tissues, but it does not appear to be significantly differentially expressed between epithelial and biphasic tissue as padj = 0.81 (padj > 0.05) (Figure 6).

According to this analysis, we did not find a statistically significant correlation between nuclear GLI1 and MPM histology (Epithelioids vs. biphasic/desmoplastic MPM) (*p* = 0.845).

## 4. Discussion

The PC is a conserved organelle protruding from the cellular membrane of all mammalian cells, involved in the regulation of many important pathways. An exhaustive understanding of all its functions is still incompletely defined and is an evolving field of research.

In cancer, PC loss is related to both pro- and anti-tumor functions that are tissue- and cancer-specific. Restoring PC functions represents an intriguing target in the field of personalized medicine.

Hh signaling is involved tissue development and homeostasis and is regulated through activators and repressors enriched in the cilium, and Hh deregulation is a feature of many cancers.

MPM is a tumor of the pleural membranes with a dismal prognosis and limited therapeutic option. For its high intra- and inter-heterogeneity, MPM cannot be considered a single tumor type, therefore requires patients-tailored therapies and reliable markers for patient stratification.

Due to the limited progress made in the last decades in MPM treatment, the repositioning of drugs already approved in the clinical practice for other tumor types represents a turning point in accelerating the introduction of novel treatments for this devastating disease [47,48]. In this regard, in the light of the encouraging results obtained with SMO/Hh inhibitors in different clinical trials, and since no direct-acting GLI1 inhibitors have entered clinical trials [38], the use of SMO-i in MPM has been investigated by many authors [37,49,50].

However, despite Hh being one of the top five deregulated pathways in MPM [45], and that GLI1 and SMO levels are correlated with a poor prognosis, the response of MPM to SMO/Hh-i is poor. While numerous in-vitro studies support a therapeutic value for SMO/Hh-i in MPM, limited information remains available from clinical studies. Two phase I solid-tumor trials, overall including five mesothelioma patients treated with the SMO inhibitors vismodegib (GDC-0449) or sonidegib (LDE225), reported no clinical benefit in MPM [49,51]. Only one case report described a durable response with vismodegib in a MPM presenting a loss-of-function mutation in PTCH1 [50]. However, mutations affecting the components that control the Hh are relatively rare in MPM compared to other SMO/HH-i resistant tumors [34,52] and cannot be considered a general mechanism of resistance.

Due to the role of Hh pathway in MPM, and the poor response to SMO/Hh inhibitors in this tumor, here, we hypothesized that Hh can be controlled by both SMO-dependent and SMO-independent mechanisms.

On these premises, and since PC is indispensable for the canonical activation of Hh pathway, here we have investigated the correlation between the presence of PC and the Hh activation in MPM tissues specimens, in non-tumoral pleural tissues, and in a panel of primary MPM cell lines.

While non-malignant mesothelial tissues retained PC, it is lost in 75% of MPM, mainly related to more aggressive phenotypes. Accordingly, the correlation between PC expression and histology is statistically significant. This observation is in line with a recent transcriptomic analysis showing increased expression of PC-related genes in epithelioid mesothelioma compared to the biphasic phenotype [53].

In agreement with IHC analysis, we observed the presence of PC in four non-tumoral mesothelial cell lines, and in four out of eight epithelioid MPM cell lines.

Then, we investigated the correlation between PC positivity and nuclear localization of GLI1 in tissues and cell cultures. Among 24 MPM specimens analyzed, 63.2% had nuclear GLI1 and, of these, 75% were PC-negative. Statistical analysis shows that the correlation between nuclear GLI1 and cilium expression is not significant supporting our hypothesis that the Hh pathway can be activated in MPM through non-canonical PC-independent pathways. We obtained similar results in primary MPM cell lines in which the absence of PC did not preclude the Hh activation.

It should be noted that although qRT-PCR analysis in PC-positive cell lines did not show high transcript levels of all Hh components, in two cell lines we observed a nuclear localization of GLI1. Although this may seem contradictory, it is in agreement with a recent analysis demonstrating that the expression levels of Hh components cannot be markers of Hh ligand-dependent activation of the pathway [54].

Furthermore, in agreement with recent studies demonstrating the synergistic effects of the simultaneous inhibition of SMO and GLI1 [32,55], it is conceivable that in PC-positive MPM the activation of the Hh signaling can be fueled by both canonical and non-canonical pathways. This could explain the poor response of MPM to SMO-i and also sustain the importance of Hh/GLI1 for MPM growth.

These results should be interpreted in the light of several limitations. First, the sample size does not allow drawing robust conclusions about the prognostic significance of PC frequency in MPM. Furthermore, in a larger series, a stratification of the morphological parameters, including nuclear pleomorphism, mitotic count, necrosis, architectural pattern, and expression of molecular markers, such as *BAP1*, *CDKN2A*, and *MTAP*, could provide useful information for the validation of these preliminary results.

It also remains to identify the non-canonical pathways that support Hh/GLI1 activation in PC-negative MPM, and their weight in the ligand-dependent regulation of the Hh signaling.

In summary, the results of this study indicate that PC loss is a frequent event in MPM, likely associated with a more aggressive phenotype, and that the Hh pathway in MPM can be activated both canonically and non-canonically. Since there is a lack of correlation between the SMO-i response and the expression of Shh and SMO upstream of the pathway [32], we sustain the importance of analyzing the presence PC, together with nuclear GLI1, as a predictive marker of response to SMO-i.

This study can help to rationalize the repositioning of Hh pathway inhibitors for MPM therapy, taking into account the high heterogeneity of this tumor.

## 5. Conclusions

The present pilot study provides the first evidence that PC loss occurs frequently in MPM and does not preclude the activation of the Hh cascade. Our findings lay the foundation for future studies in the context of MPM, highlighting once again the need of patient-tailored therapies for this cancer.

## Figures and Tables

**Table 1:** PC expression in FFPE specimens. (*n*) = number of patients; mix = intra-heterogeneity in PC expression.

Specimens’ Histotype	PC Status (%)
Epithelioid (*n* = 19)	Negative: 42.1% (*n* = 8) Positive: 36.8% (*n* = 7) Mix= 21.0% (*n* = 4)
Biphasic (*n* = 4) and desmoplastic (*n* = 1)	Negative = 100%
Pleuritis (*n* = 2) and reactive mesothelial hyperplasia (*n* = 2)	Positive = 100%

**Table 2:** Expression of PC in non-tumoral mesothelial and in mesothelioma cell lines. PC expression was analyzed by IF with Arl13b antibody.

**Pc-Positive (%) Non-Malignant Mesothelial Cells**	
HMC13	34.7
HMC12	46.7
HMC7	40.3
LP-9	68.6
**PC-Positive (%) MPM Cells**	
MMP1	0%
MMP4	0%
MMP14	0%
MMP18A	46.8%
MMP21	71.3%
MMP23	80.2%
MMP32	23.8%
MMP43	0%

**Table 3:** IHC analysis of GLI1 expression in MPM. *n* = sample size.

GLI1 Score	PC Positive (%) (*n* = 5)	PC Negative (%) (*n* = 14)
0	40	35.7
1	0	7.1
2	20	35.7
3	40	21.4

## Data Availability

The data that support the findings of this study are available from the corresponding author upon reasonable request.

## Supplementary Materials

The following supporting information can be downloaded at: https://www.mdpi.com/article/10.3390/cancers14215216/s1, Table S1: Clinical information about mesothelial and mesothelioma patient-derived cell lines; Table S2: Clinical information about non-malignant and mesothelioma FFPE tissues; Table S3: Top five pathways deregulated in mesothelioma; Table S4: qRT-PCR analysis of *GLI1*, *PTCH1* and *c-MYC* genes; Figure S1: TEM images of primary mesothelioma cells.

## Abbreviations

PC: Primary Cilium; Hh: Hedgehog; MPM: Malignant Pleural Mesothelioma; SMO: G-protein-coupled receptor smoothened; GLI: Glioma-associated oncogene-transcription factors; PTCH: Patched homolog.
