# Supplementary material for: Analysis of Primary Cilium Expression and Hedgehog Pathway Activation in Mesothelioma Throws Back Its Complex Biology
Source: Cancers (Basel). 2022 Oct 25;14(21):5216. doi: 10.3390/cancers14215216 (PMC9654223; doi:10.3390/cancers14215216)
Supplement: Supplementary file 1 [file cancers-14-05216-s001.zip › cancers-1931731-supplementary final.pdf]

Cell line	Age	Gender	Diagnosis	Chemotherapy (platinum and anti-folate)
MMP1	77	Male	Poorly differentiated epithelioid	Yes
MMP4	72	Male	Epithelioid	No
MMP14	79	Male	Biphasic	Yes
MMP18	72	Female	Early stage epithelioid	Yes
MMP21	74	Male	Early stage epithelioid	Yes
MMP23	67	Female	Epithelioid	Yes
MMP32	68	Male	Epithelioid	Yes
MMP43	69	Male	Epithelioid	Yes
HMC7	16	Male	Mesothelial hyperplasia	n.a.
HMC12	61	Male	Mesothelial hyperplasia	n.a.
HMC13	73	Male	Pleuritis	n.a.

Supplementary Table S1. Clinical information about mesothelial and mesothelioma patient-derived cell lines. n.a.= not applicable

n° of patients	Gender	Diagnosis	Stage at diagnosis	Treatment	Overall Survival
18	Female (n=2);<br>Male (n=17)	16 epithelioids<br>4 biphasic, 1 desmplastic	14 patients: IB<br>2 patients: II<br>2 patients: IIIA	CHT (platinum plus pemetrexed= = 17	21,952 ± 2,246 months
4	Male	Non-malignant mesothelial reactive hyperplasia (n=2); pleuritis (n=2)	n.a.	n.a.	

Supplementary Table S2. Clinical information about non-malignant and mesothelioma FFPE tissues. CHT: Chemotherapy; n.a.: not applicable

Pathway Name	Average Number of Perturbations
Nephrin/Neph1 signaling in the kidney podocyte	18
Ephrin A reverse signaling	13
Syndecan-1-mediated signaling events	11
Signaling events mediated by the Hedgehog family	10
IL4-mediated signaling events	10

Supplementary Table S3. Top five pathways deregulated in mesothelioma.
https://gdac.broadinstitute.org/runs/analyses__2014_10_17/reports/cancer/MESO-TP/Pathway_Paradigm_RNASeq_And_Copy_Number/nozzle.html

**Cell line**	***GLI1* Fold induction ± St. Dev.**	***PTCH1* Fold induction ± St. Dev.**	***c-MYC* Fold induction ± St. Dev.**
HMC7	1	1	1
LP-9	0.75±0.05	1.16±0.86	0.88±0.22
MMP1	1.85±0.33	3.81±0.97	1.65±0.18
MMP4	5.73±1.92	3.73±0.89	2.19±0.43
MSTO-211H	7.96±1.56	4.24±0.98	3.04±0.57
MMP18	1.96±0.70	0.84±0.49	0.96±0.1
MMP21	0.94±0.17	1.92±0.79	0.69±0.09
MMP23	2.87±0.86	2.25±0.6	1.1±0.2
NCI-H2052	1.51±0.53	1.18±0.11	2.01±0.36

Supplementary Table S4. qRT-PCR analysis of *GLI1*, *PTCH1* and *c-MYC* genes. Relative fold induction of the analyzed genes vs HMC7 normal mesothelial cells. St. Dev.= Standard Deviation.

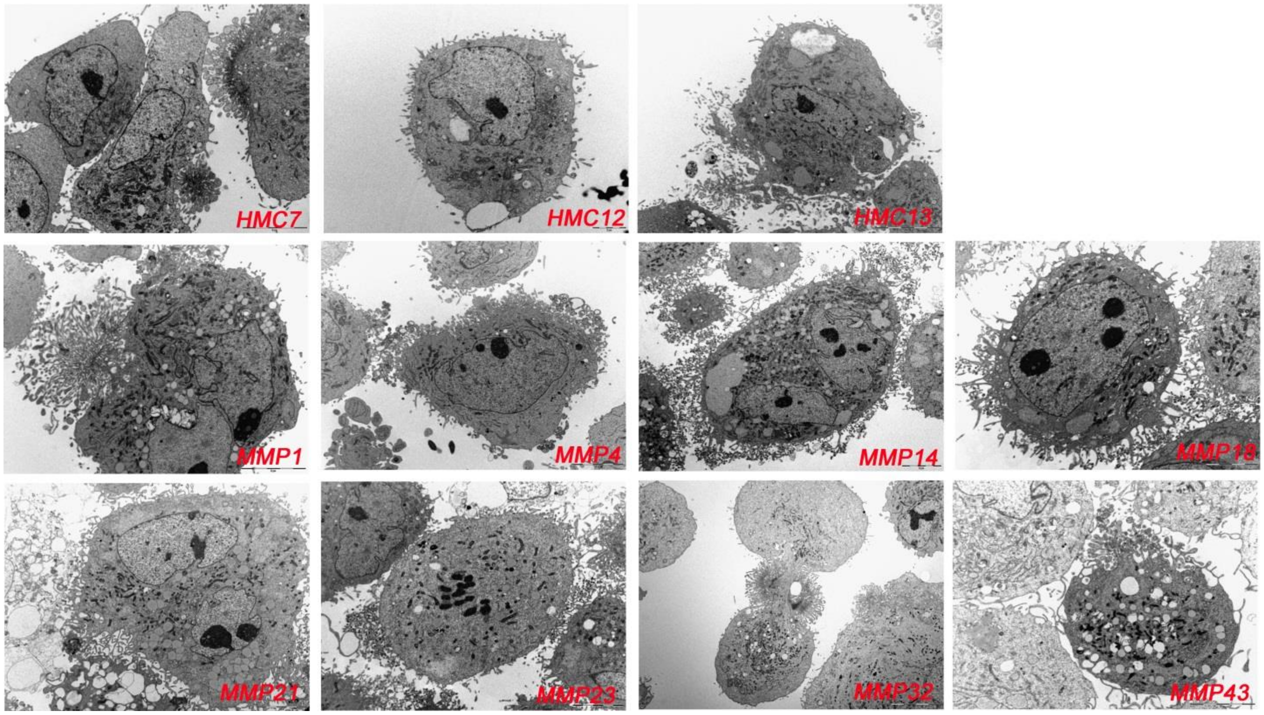

Supplementary Figure S1. TEM images of primary mesothelioma cells. The mesothelial cells are covered by abundant, long, frequently branching microvilli (original magnification 3500x).
